# Supplementary material for: Time‑resolved multi-omic analysis of paclitaxel exposure in human iPSC‑derived sensory neurons unveils mechanisms of chemotherapy‑induced peripheral neuropathy
Source: Cell Death Dis. 2026 Feb 10;17(1):211. doi: 10.1038/s41419-026-08445-2 (PMC12921266; doi:10.1038/s41419-026-08445-2)
Supplement: Supplementary file 15 — Dataset 1. [file 41419_2026_8445_MOESM15_ESM.zip › CDD_Open_Data/04_RNA_Seq/Timeline_panel_plots_etc_2023-01-23_1609.zip!/Panel_Plots_etc/Evidence_Plots/post5d/evidence_plot_standard contrasts_msigdb_reactome_5d_PTX_vs_DMSO_ID6_M27934_REACTOME_ASSEMBLY_AND_CELL_SURFACE_PRESENTATION_OF_NMDA_RECEPTORS.pdf]

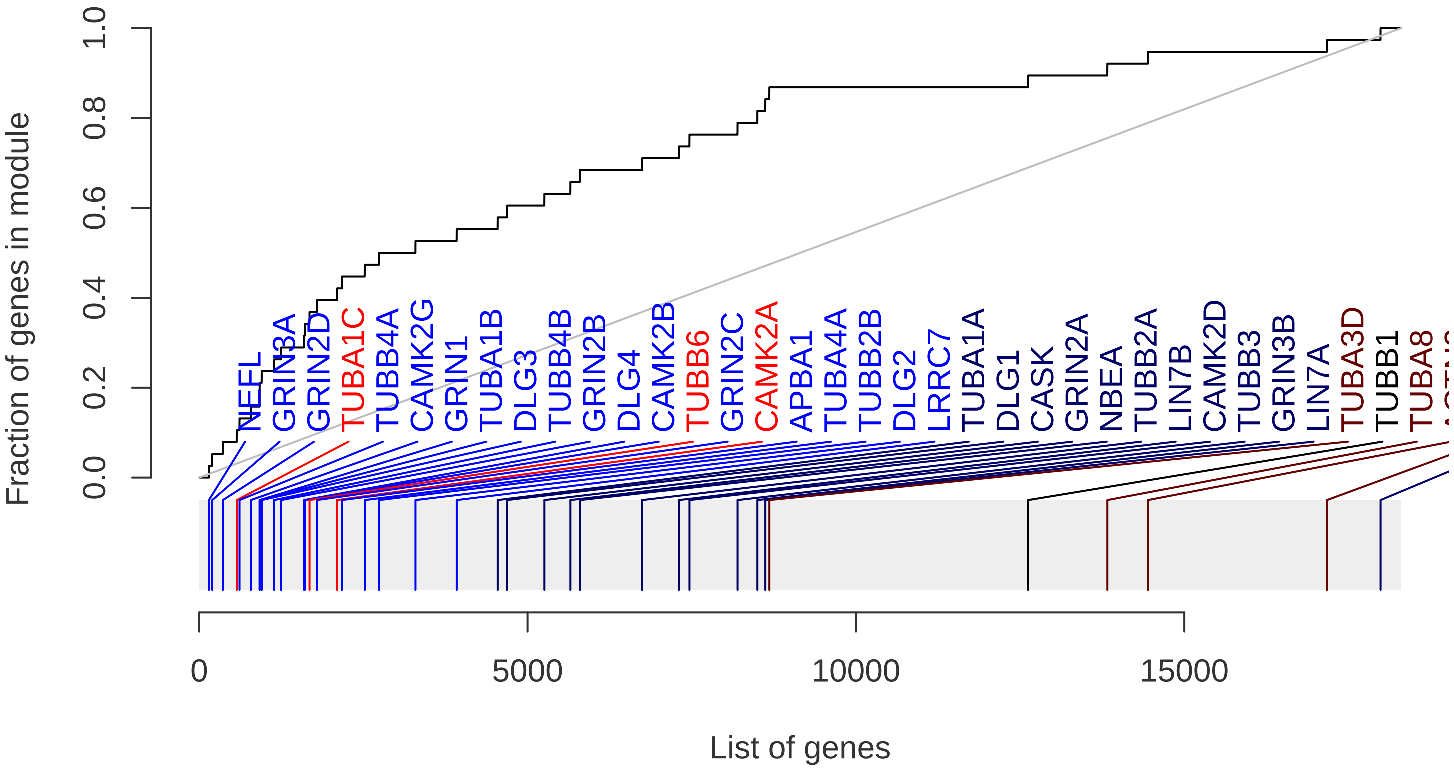

Fraction of genes in module
0.0
0.2
0.4
0.6
0.8
1.0
NEFL
GRIN3A
GRIN2D
TUBA1C
TUBB4A
CAMK2G
GRIN1
TUBA1B
DLG3
TUBB4B
GRIN2B
DLG4
CAMK2B
TUBB6
GRIN2C
CAMK2A
APBA1
TUBA4A
TUBB2B
DLG2
LRRC7
TUBA1A
DLG1
CASK
GRIN2A
NBEA
TUBB2A
LIN7B
CAMK2D
TUBB3
GRIN3B
LIN7A
TUBA3D
TUBB1
TUBA8
0
5000
10000
15000
List of genes
